# Effectiveness of an integrated hospital-community care pathway for fragility fractures’ secondary prevention: the PROMOTER-II study

**DOI:** 10.1007/s11657-025-01642-0

**Published:** 2025-12-16

**Authors:** Laura Bogliolo, Silvia Grignaschi, Catherine Klersy, Pietro Perotti, Simona Dalle Carbonare, Silvia Balduzzi, Michela Milanesi, Giuseppe Giuffrè, Carmelinda Ruggiero, Maria Cristina Caffetti

**Affiliations:** 1 Rheumatology Unit, Fondazione IRCCS Policlinico San Matteo, Pavia, Italy; 2 Rehabilitation Unit, Ospedale Unificato di Broni-Stradella, ASST Pavia, Pavia, Italy; 3 Biostatistics & Clinical Trial Center, Research Department, Fondazione IRCCS Policlinico San Matteo, Pavia, Italy; 4 ATS Pavia, Pavia, Italy; 5 Primary Care Division, ATS Pavia, Pavia, Italy; 6 Geriatric Section, Department of Medicine and Surgery, University of Perugia, Perugia, Italy

**Keywords:** Osteoporosis, Fragility fracture, Fracture liaison services, Fracture treatment, Hospital-community pathway

## Abstract

***Summary*:**

As the global population ages, osteoporosis and fragility fractures (FF) are emerging health concerns worldwide. This study assesses the effectiveness of an integrated hospital-community pathway in improving prescription and adherence to anti-fracture treatment. Additionally, it analyzes the re-fracture rate and mortality at a 24-month follow-up from an index FF.

**Purpose:**

Evaluate the effectiveness of an integrated healthcare pathway (IHCP) on appropriateness and adherence to anti-fracture treatment (AFT) in adults aged ≥ 50 years, hospitalized due to fragility fracture (FF). Re-fractures and mortality rates were explored as secondary outcomes.

**Methods:**

Subjects aged 50 years and more, resident in the province of Pavia, Italy, were enrolled at the time of hospital admission due to major FF, in the period 2016–2018 and 2019–2020 before and after the implementation of the IHCP, respectively. Data were extracted from the administrative database of the Health Protection Agency of Pavia, starting from the index event and until 24 months. Data analyses were conducted for the primary and secondary outcomes. Univariate and multivariate Cox regression models were fitted.

**Results:**

Among 9186 participants (74.7% women), aged 78 years, 12.7% initiated an AFT, and half of them (6.3%) within 6 months after the index FF. Comparing pre- and post- IHCP implementation phases, the appropriateness of AFT initiation increased in subjects with index humerus/wrist FF, oral AFT prescription decreased, while subcutaneous or intravenous treatments increased (*p* < 0.001). Total adherence to AFT slightly increased. The re-fracture rate was 5.2 per 100 person/year, with age, IHCP implementation, and hip FF associated with a higher likelihood of refracture. The mortality rate was 13.4 per 100 person/year, with age, male gender, IHCP, and hip fracture as independent risk factors. Appropriate AFT significantly reduced mortality compared to no treatment (HR 0.55), with one-year adherence showing the strongest benefit (HR 0.18). Adherent patients had threefold lower mortality than non-adherent ones. Hip and vertebral fractures had an increased death risk (*p* < 0.001) compared to wrist and/or humerus. AFT results in an effect modifier of the association between the site of FF and mortality risk (*p* = 0.003).

**Conclusion:**

The implementation of an IHCP, based on a FLS integrated model, shows a tendency to improve FF management, especially among younger patients with humerus or wrist FF. We confirmed a lower mortality among subjects received appropriate AFT.

**Supplementary Information:**

The online version contains supplementary material available at 10.1007/s11657-025-01642-0.

## Introduction

Osteoporosis (OP) is a chronic disease causing macro and micro-architectural changes in the bone tissues, reduction of resistance, and predisposing to a high risk of fractures, both spontaneous and due to low-energy trauma [1, 2]. Fragility fractures (FFs) can occur at multiple sites, including vertebrae, wrist, femur, and humerus. The risk of recurrent FFs exponentially increases depending on the number of previous fractures [3, 4].

FFs also represent a major cause of individuals’ disability and mortality, adding a significant economic burden to National Health Systems (NHS) [5]. The OP scorecard (SCOPE 2021) reported that more than 23 million subjects are at high risk of FFs in Europe, and 4.3 million fractures cost the NHS 56 billion euros each year [6]. Among FFs, hip fracture is burdened with a death rate of 105 subjects every 100.000 and causes a significant reduction in patients’ quality of life [7]. With the aging of the population, the incidence of FFs is projected to increase by 23.4% in 2034 [6].

Accordingly, to SCOPE 2021 [6], in Italy, the prevalence of OP was estimated to be about 6.3%, and about 71% of individuals with OP do not receive appropriate treatments and care. However, the International Osteoporosis Foundation (IOF) data are probably underestimated. In 2019, the Italian NHS counted 2.060 fractures every 100.000 inhabitants [6], leading to an economic burden estimated at 9.45 billion euros per year [8], with costs per person increasing by 21%–34% from 2010 to 2019 [6, 7].

Patients with FFs deserve proper attention in a specific diagnostic and therapeutic framework. The Fracture liaison service (FLS) is a well-established model of care designed and validated for the prevention of FFs, rehabilitating and treating complications from FFs [9, 10]. In Italy, since 2016, an NHS resolution has recommended the regional development of an integrated, multidisciplinary diagnostic and therapeutic healthcare pathway (IHCP) for the management of chronic diseases, including OP and FFs. The hospitals and the community care of Pavia’s district established a dedicated hospital-community interdisciplinary diagnostic and therapeutic care pathway [11]. The Pavia’s IHCP was developed according to the FLS model [12] and aligned with the Italian Drug Agency (AIFA)’s resolution for primary and secondary prevention of FFs [13], which defines the rules for the reimbursement of OP drugs. The IHCP was developed as a tool to identify patients at high risk of FFs and ensure their appropriate management and care according to the severity and complexity of the disease.

Using a pre- and post-intervention design, this study aims to evaluate the effectiveness of the IHCP in capturing, initiating appropriate treatments, and monitoring adults and older adults aged 50 years and older, hospital-admitted due to major FFs in the Pavia district. Specifically, we sought to assess the impact of the IHCP on the appropriate treatment initiation rate and 1-year adherence to anti-fracture treatments (AFT), including vitamin D and specific OP drugs. We also evaluate the impact of IHCP implementation on adverse outcomes, including 2-year subsequent FFs and mortality from the index event.

## Methods

### Study design

The PROMOTER-II (PRogetto Osteoporosi Multidisciplinare Ospedali e TERritorio – secondary prevention) study is a multicenter pre- and post-intervention observational study aimed at improving secondary prevention in subjects who suffered major osteoporotic FFs in the province of Pavia [11], which encompasses 536,406 citizens, with 49.1% aged 50 years and more. The pre-intervention phase was conducted from 2016 to 2018, while the post-intervention phase was conducted from 2019 to 2021.

**Fig. 1:**
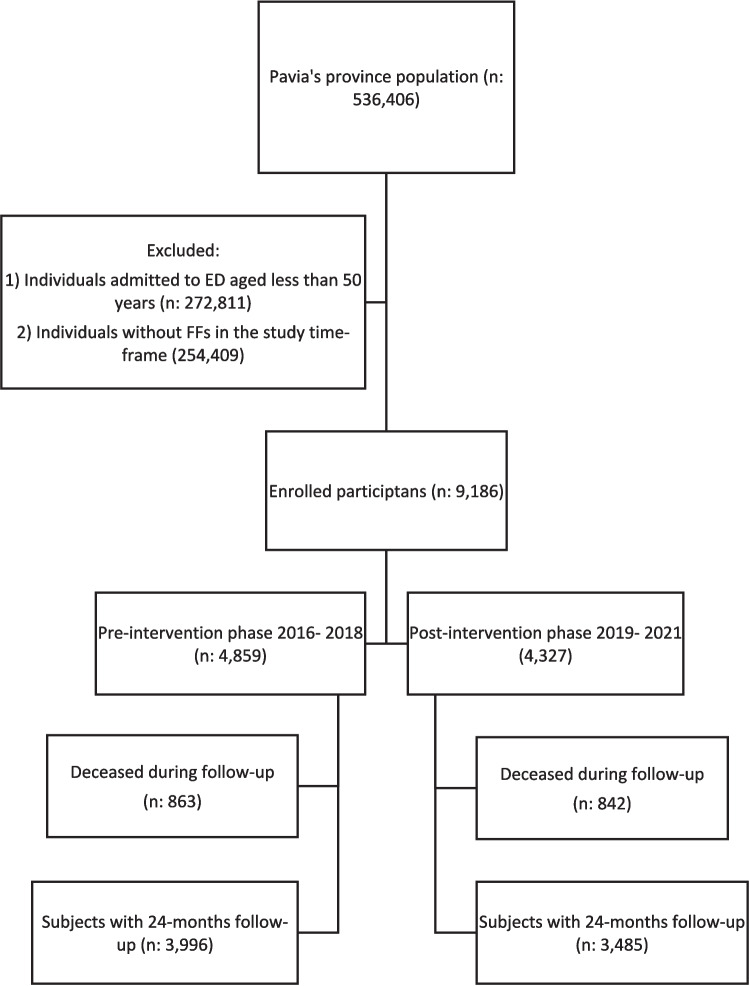
Diagram of the study population. Legend: FF, fragility fracture; IHCP, integrated health-care pathway

Eligible participants were men and women aged ≥ 50 years old, residents of the province of Pavia, who were admitted due to low-energy major FF (i.e., hip, vertebral, humerus, or wrist) to the Emergency Department (ED) or the Trauma and Orthopedic Units of Pavia, Stradella, Vigevano, and Voghera hospitals. Participants were identified based on the International Classification of Diseases (ICD)−9CM. Specifically, ICD codes 805* for vertebral fractures and 820* for hip fractures, code 812* for humerus fractures, and 814* for wrist fractures, or ICD-9CM 733.1* for the same fracture sites (733.13, 733.14, 733.11, 733.12). All subjects reported fractures after low-energy trauma; people who underwent high-energy accidents or trauma were excluded. All participants underwent a 24-month follow-up starting from the index event. Data on the major clinical outcomes, including death and recurrent FFs, were collected using administrative databases and presented using the flow diagram in Fig. 1. The study was conducted in accordance with the Helsinki Declaration and was approved by the local institutional ethics committee (IEC Code 20210047334).

### Intervention

The intervention consisted of implementing an IHCP, established by a resolution of the local healthcare authorities and released in January 2019. The resolution was the result of several multi-professional meetings, conducted with the primary objective of training, networking, and strengthening the GP’s ability to identify and investigate high-risk subjects, thereby allowing bone specialists (BSs) to establish a pathway for high-risk patients to receive appropriate care [11]. The implementation phase, which lasted 1 month, was conducted in January 2019. During this period, health authorities convened BSs, general practitioners (GPs), and other medical specialists to align and solve issues related to the operationalization of the care pathway. The overall protocol, the choice of AFT to initiate, and the follow-ups were established according to best clinical practice, the AIFA 79 resolution [13], the SIOMMMS (Italian Society of Osteoporosis and Mineral Bone Diseases), and international osteoporosis guidelines [10, 14]. Briefly, reimbursement of OP drugs depends on the balance between benefits and risks, which is complex at both individual and societal levels, and includes pharmaco-economic aspects. Therefore, OP treatment is covered by the NHS for patients with high fracture risk, where the Number Needed to Treat justifies long-term therapy and prioritizes secondary prevention, especially in patients with prior vertebral or femoral fractures, low bone density, or steroid therapy based on evidence from major studies. Vertebral fractures are diagnosed with Genant criteria [15], while severe or moderate fractures are emphasized for romosozumab eligibility [13].

### Study variables and indicators

Demographics were collected about age and sex. Clinical data included type and site of the first FF causing ED or hospital admission, based on ICD-9-CM codes, then type of AFT prescribed, including alendronate, risedronate, zoledronate, denosumab, teriparatide, and vitamin D. Data on recurrent major fractures and mortality were captured within 24 months from the index fracture by exploring Pavia’s administrative database of the Health Protection Agency (ATS). The last check of the administrative database was performed on 31 st December 2023.

AFT appropriateness was defined as the prescription of specific antiresorptive or anabolic drugs, independent of vitamin D and calcium supplements, performed within 6 months from the index FF. AFT adherence was defined as ongoing treatment after 1 year from the index prescription, with a daily drug dosage ≥ 80%.

### Primary and secondary outcomes

The primary outcomes are the appropriateness of AFT initiation and 1-year adherence to AFT, as compared between participants in the pre- and post-intervention phases. Secondary outcomes aim to compare rates of subsequent major FFs causing ED or trauma ward admission and mortality rates between participants enrolled in the pre- and post-intervention phases.

### Data extraction and statistical analysis

Data for the present study were extracted from Pavia’s administrative databases. Annual data from 2016 to 2018 (pre-intervention) and from 2019 to 2021 (post-intervention) were retrieved; meanwhile, follow-up data were retrieved up to December 2020 (pre-intervention) and December 2023 (post-intervention) (Fig. 1).

With a sample size of over 4200 subjects from every time-frame (2016–2018 and 2019–2021), we may elicit an effect size of 4% and 5%, respectively, for appropriateness and adherence when comparing the rates in both time frames, with a 90% power and a conservative type I error of 1 per 1000. The rate of appropriateness before the IHCP adoption is assumed to be 20%, and the rate of adherence is 50% according to AIFA records. The rate of appropriateness before the IHCP adoption is assumed to be 20%, and the rate of adherence is 50% according to AIFA records.

To reduce the risk of duplication bias, we removed from the database the IDs that had two or more hospital admissions within 60 days, considering as a single admission those for surgery following a first access for the same fracture in the emergency room.

Continuous variables were described using the mean and standard deviation (SD), and categorical variables were presented as counts and percentages. We used the Fisher exact test to compare them between time frames and the test for trend to assess changes over the years. For the analysis, age was categorized into quartiles. Estimates of appropriateness and adherence, along with their 95% confidence intervals (95% CI), were calculated. Generalized linear models extended to the binomial family were used to compare the two time frames for both appropriateness and adherence, while adjusting for the site of index fracture. The risk difference and 95%CI were computed. Sensitivity analysis of these two endpoints was performed after the removal of data gathered in 2020. The interaction of time frame and type of index FF was assessed. Cumulative (event-free) survival was computed and plotted using the Kaplan Meier. Curves were compared using the log-rank test. Rates per 100 person-year and 95%CI were calculated. Univariate and multivariable Cox regression models were fitted to assess the role of AFT, AFT appropriateness, adherence to treatment, age, and sex, as well as the impact of intervention on re-fracture rate and mortality. For the purpose of the analysis, treatment appropriateness was categorized into 4 levels, i.e., untreated, inappropriately treated (those who receive AFT after 6 months from index event), appropriately treated, and appropriately and adherently treated. The Harrell’s c statistic was computed for model discrimination. The proportional hazard assumption was assessed graphically. A predefined subgroup analysis explored the prognostic role of the site of index FF in untreated and treated participants. A two-sided *p*-value < 0.05 was considered statistically significant. The Stata software (release 18, StataCorp, College Station, TX, USA) was used for computation.

## Results

### Main characteristics of the entire sample

Overall, 9186 participants were enrolled in the study at the time of ED or Trauma ward admission: 4859 from January 2016 to December 2018 (the pre-intervention phase) and 4327 from February 2019 to December 2021 (the post-intervention phase, Fig. 1). The majority of participants (*n* 6858; 74.7%) were women, with an average age of 78 years (SD 11.4).

Overall, hip FFs were more prevalent in subjects older than 70 years, whereas humerus or wrist FFs were more prevalent in those younger (*p* < 0.001). Specifically, participants with hip fractures (*n* 4223; 46%) were on average 82.3 ± 9.4 years old, with an annual incident fracture rate of 0.79 per 100 person-years and a mortality incidence rate of 0.21 per 100 person-years. Participants with humerus or wrist fractures (*n* 3186; 34.7%) were 73.8 ± 11.6 years old, with an annual incident fracture rate of 0.59 per 100 person-years and a mortality incidence rate of 0.06 per 100 person-years. Participants with vertebral fractures (*n* 1768; 19.3%) were 75.2 ± 11.5 years old on average, with an annual incident fracture rate of 0.33 per 100 person-years and a mortality incidence rate of 0.05 per 100 person-years. From 2016 to 2021, the distribution of FF types remained balanced over time (*p* = 0.286).

After the index fracture, only 1164 (12.7%) participants initiated an AFT in the entire sample, with an appropriateness rate of 49.7% (*n* 578). Specifically, the initiation rate was 11.7% (*n* 374) after humerus or wrist fracture, 21.3% (*n* 377) after vertebral fracture, 9.8% (*n* 413) after hip fracture. Only 1078 participants (11.7% of the entire sample) started a complete treatment with AFT plus vitamin D, with an appropriateness rate of 42%.

Among participants who appropriately initiate treatment, 311 (53.8%) remained adherent to AFT, and 180 (39.7%) completed treatment after 1 year from the initiation time. About 8.2% (*n* 749) initiated oral AFT, 3.4% (*n* 317) subcutaneous AFT, 1.1% (*n* 98) intravenous AFT, and 50% (*n* 4610) received vitamin D supplementation. In the overall cohort, one out of three subjects (*n* 3532; 38%) received only vitamin D supplementation.

After 24 months from the index FF, we observed 633 (6.9%) re-fractures, corresponding to a re-fracture rate of 5.2 per 100 person-years (95% CI 4.8–5.7). Specifically, there were 364 re-fractures (57.5%) following hip fracture, 167 (26.4%) after humerus or wrist fracture, and 102 (16.1%) subsequent vertebral FF. During the follow-up, 1705 (18.6%) participants died, showing an overall mortality rate of 13.4% (95%CI 12.7–14.0), mostly among participants who suffered hip fracture (*n* 1144; 67.1%), then (*n* 305; 17.9%) among those who experienced humerus or wrist fracture, and lastly (*n* 256; 15%) in those with vertebral fracture.

### AFT appropriate initiation and 1-year adherence

Table 1 reports appropriateness rates to AFT alone or in combination with vitamin D (Table 1, parts A and B), and 1-year adherence rates to appropriate treatments (Table 1, parts C and D) by comparing pre- and post-intervention phases.

Compared to the pre-intervention phase, a tendency to higher rates of appropriate initiation of AFT (47.3% vs 52.4%; *p* = 0.088) was found in the post-intervention phases, with a risk difference of 5.1% (95% CI −0.6–10.9). In a sensitivity analysis, where year 2020 was removed, rates of appropriate initiation of AFT were 47.3% and 54.2%, respectively, with a risk difference of 6.9% (95%CI 0.6–12.3), very close to the original value. Supplementary Fig. 1, panel a, shows the yearly rate to slightly decrease in 2020 and increase in 2021.

We observed that the type of index FF acting as an effect modifier (*p* for interaction 0.046) (Table 1, part A) and appropriateness was higher mostly in the humerus/wrist group after the implementation phase (Supplementary Fig. 1bto). The initiation rates of complete treatment increased in a statistically significant manner (39.1% vs 45.5%; *p* = 0.041) in the post-intervention phase, with a risk difference of 6.3% (95% CI 0.4–12.2). We again confirmed an effect modifier of the association due to the type of fracture (Table 1, part B) and again appropriateness was higher mostly in the humerus/wrist group after the implementation phase (Supplementary Fig. 2).

**Table 1:** Appropriateness and adherence to AFT and AFT plus vitamin D in participants enrolled in the pre- and post-intervention phases

**A**	**AFT Appropriateness** ***N***=**578**	**Pre-intervention** **N (% appropriate of all treated)**	**Post-intervention** **N (% appropriate of all treated)**	**Difference (95%CI)**	***p***-**value**
All		300 (47.3%)	278 (52.4%)	5.1 % (-0.6 to 10.9)	0.088
By index fracture type (p for interaction 0.046)					
Humerus / wrist (N=142)		60 (30.0%)	82 (47.1%)	17.1 % (7.5 to 26.9)	<0.001
Vertebra (N=213)		122 (56.7%)	91 (56.2%)	-0.6 % (-10.7 to 9.5)	0.917
Hip (N=223)		118 (53.9%)	105 (54.1%)	0.2 % (-9.4 to 9.9)	1.00
**B**	**AFT+vitamin D Appropriateness** ***N***=**453**				
All		231 (39.1%)	222 (45.5%)	6.3% (0.4 to 12.2)	0.041
By index fracture type (p for interaction 0.026 )					
Humerus / wrist (N=103)		39 (21.1%)	64 (40.2%)	19.2% (9.5 to 28.9)	<0.001
Vertebra (N=160)		91 (45.5%)	69 (46%)	0.5% (-10.0 to 11.0)	1.00
Hip (N=190)		101 (49.3%)	89 (49.7%)	0.4% (-9.6 to 10.5)	1.00
**C**	**AFT Adherence** ***N***=**311**	**Pre-intervention** **N (% adherent of all appropriate)**	**Post-intervention** **N (% adherent of all appropriate)**	**Difference (95%CI)**	***p*-value**
All		159 (53%)	152 (55%)	1.7 % (-0.6 to 9.8)	0.738
By index fracture type (p for interaction 0.146)					
Humerus / wrist (N=65)		24 (40%)	41 (50%)	10.0% (-6,5 to 26.5)	0.306
Vertebra (N=115)		69 (57%)	46 (51%)	-0.6% (-19.5 to 7.5)	0.407
Hip (n=131)		66 (56%)	65 (62%)	6.0% (-6.9 to18.9)	0.414
**D**	**AFT+vitamin D Adherence** ***N***=**180**				
All		78 (33.8%)	102 (45.9%)	12.2 (3.2 to 21.1)	0.009
By index fracture type (p for interaction 0.121)					
Humerus / wrist (N=39)		10 (25.6%)	29 (45.3%)	19.7% (1.3 to 38.0)	0.060
Vertebra (N=70)		40 (44.0%)	30 (43.5%)	-0.4% (-16.0 to 15.0)	1.00
Hip (n=71)		28 (27.7%)	43 (48.3%)	20.6% (7.0 to 34.2)	0.004

*AFT* anti-fracture treatment, *IHCP* integrated health-care pathway, *CI* confidence interval

Notably, a higher prescription of AFT to subjects with humerus or wrist FFs drove the increase in appropriate initiation rates in the entire sample (Table 1, part A, and Supplementary Fig. 1, panel b, test for trend *p* < 0.001). The appropriate AFT initiation rate increased by 17% (95%CI 7.5–26.9) in humerus or wrist index FFs and that of complete treatment by 19.2% (95%CI 9.5–28.9) (Table 1, A and B parts, and Supplementary Fig. 2, panel b). There were no changes in the initiation rates of subjects affected by vertebra and hip index FFs (Table 1, parts A and B, and Supplementary Fig. 1 and 2, panel c and d, test for trend *p* = 0.470 and *p* = 0.500, respectively).

Compared to the pre-intervention phase, the prescription and initiation of oral AFT decreased in the post-intervention phase (70% vs 58%; *p* < 0.001), while we observed an increase for the subcutaneous (24% vs 31%; *p* = 0.01) and intravenous (6% vs 11%; *p* = 0.006) treatment prescription (data not shown).

Rates of 1-year adherence to AFT remained similar (53% vs 55%) in both phases without signal for effect modification due to type of index FF (*p* for interaction 0.146) (Table 1, part C). The adherence rates showed fluctuations over time, without statistically significant changes in the entire sample (Supplementary Fig. 3, panel a, test for trend, *p* = 0.055), neither by type of index FFs. A tendency toward a positive trend in adherence rates was identified in the period 2019–2021 among subjects with humerus or wrist (Supplementary Fig. 3, panel b, test for trend *p* = 0.102) and vertebra (panel c, test for trend *p* = 0.602) index FFs, while the trend remained even more blunted among subjects with hip index FFs (panel d, test for trend *p* = 0.107).

The 1-year adherence to appropriate and complete treatment showed a statistically significant increase (33.8% vs 45.9%; risk difference 12.2%, 95% CI 3.2–21.1; *p* = 0.009) in the post- compared to the pre-intervention phase, without an effect modifier due to the type of index FFs over the period (*p* for interaction 0.121; Table 1, part D, and Supplementary Fig. 4, panel a). In a sensitivity analysis, where year 2020 was removed, rates of adherence to appropriate and complete treatment were 38.1 and 44.7% respectively, with a risk difference of 10.9% (95%CI 1.3–20.6), close to the original value. Supplementary Fig. 4, panel a, shows the yearly rate to progressively increase after 2019.

Adherence to appropriate and complete treatment increased especially among participants with humerus and wrist index FFs (25.6% vs 45.3%; risk difference 19.7%, 95% CI 1.3–38; *p* = 0.06; Supplementary Fig. 4, panel b; test for trend *p* < 0.001), and those with hip index FFs (27.7% vs 48.3%; risk difference 20.6%, 95% CI 7–34.2; *p* = 0.004; Supplementary Fig. 4, panel d; test for trend *p* 0.030).

### Recurrent fragility fractures

Re-fracture rate was 5.2 per 100 person-years (95% CI 4.8–5.7), with older age, intervention implementation, and site of index FFs acting as independent predictors (Table 2). Specifically, compared to subjects with humerus or wrist FFs, those with hip fractures experienced a significantly higher risk of re-fracture (HR 1.64, 95%CI 1.35–1.98, *p* < 0.001), which was also confirmed against participants with vertebral fractures (*p* = 0.001). The IHCP implementation was associated with a higher likelihood of re-fracture (*p* < 0.001). Participants who received inappropriate treatments experienced a higher re-fracture risk (HR 1.34, 95%CI 1.00–1.80; *p* = 0.048) compared to those who were untreated. No signal for an effect modification of the association between treatment and re-fracture was found for other covariates included in the analysis.

**Table 2:** Cox model for refracture-free survival and survival over a 24 months follow-up

Variable	Refractures rate per 100 person-year (95%CI)	Refractures Multivariable model Model chi^2^(10) 107.94, *p* < 0.001 Harrell’s *C* 0.62		Mortality rate per 100 person-year (95%CI)	Mortality Multivariable model Model chi^2^(10) 1323.68, *p* < 0.001 Harrell’s *C* 0.74	
	*N* = 5.2 per 100 *p*/year (4.8–5.7)	HR (95%CI)	*p*-value	*N* = 13.4 per 100 *p*/year (12.7–14)	HR (95%CI)	*p*-value
**Age**			**< 0.001**			**< 0.001**
< = 71	2.7 (2.2–3.3)	1		3.1 (2.6–3.7)	1	
72–80	5.6 (4.9–5.6)	1.83 (1.43–2.36)	< 0.001	8.8 (7.8–10.0)	2.70 (2.17–3.36)	< 0.001
81–87	6.6 (5.7–7.6)	2.00 (1.56–2.58)	< 0.001	15.4 (14.1–17.0)	4.15 (3.37–5.11)	< 0.001
> 87	6.7 (5.8–7.8)	1.80 (1.38–2.34)	< 0.001	30.2 (28.2–32.4)	7.06 (5.77–8.65)	< 0.001
**Sex**			**0.310**			**< 0.001**
F	5.4 (4.9–5.9)	1		11.6 (10.9–12.3)	1	
M	4.7 (4.0–5.6)	0.90 (0.75–1.10)		19.1 (17.6–20.7)	1.78 (1.61–1.98)	
**IHCP implementation**			**< 0.001**			**0.046**
Before	4.5 84.0–5.0)	1		12.7 (11.9–13.6)	1	
After	6.1 (5.5–6.8)	1.34 (1.15–1.57)		14.1 (13.2–15.1)	1.10 (1.00–1.21)	
**Treatment**			**0.139**			**< 0.001**
Untreated	5.2 (4.8–5.7)	1		15.2 (14.5–16.0)	1	
Inappropriately	6.0 (4.6–7.8)	1.34 (1.00–1.80)	0.048	0.4 (0.2–1.1)	0.05 (0.02–0.14)	< 0.001
Appropriately	3.9 (2.3–6.5)	0.81 (0.48–1.35)	0.416	6.2 (4.2–9.2)	0.55 (0.37–0.82)	0.003
Appropriately and adherent	4.3 (3.0–6.7)	0.85 (0.54–1.34)	0.487	2.1 (1.1–3.8)	0.18 (0.10–0.34)	< 0.001
**Site of index FF**			**< 0.001**			**< 0.001**
Humerus/wrist	3.6 (3.1–4.2)	1		6.4 (5.7–7.1)	1	
Vertebra	4.2 (3.4–5.1)	1.13 (0.88–1.45)	0.349	10.1 (8.9–11.4)	1.40 (1.19–1.66)	< 0.001
Hip	7.2 (6.5–8.0)	1.64 (1.35–1.98)#	< 0.001	21.0 (19.8–22.3)	1.98 (1.74–2.25)#	< 0.001
		*#hip vs vertebra p* = *0.001*			*#hip vs vertebra p* < *0.001*	

*IHCP* integrated health-care pathway, *FF* fragility fracture, *HR* hazard ratio, *CI* confidence interval

### Mortality

**Fig. 2:**
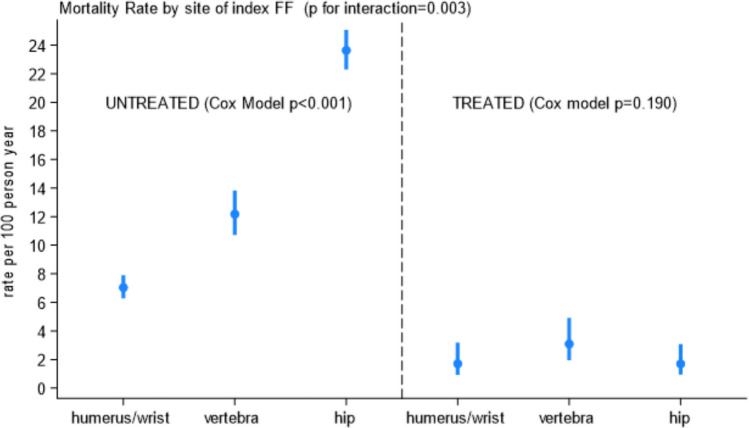
Mortality rate by site of index FFs and survival analysis based on Cox models between untreated and treated high-risk subjects. Untreated subjects: vertebra vs. humerus or wrist: HR 1.71, 95%CI 1.44–2.03; hip vs. humerus/wrist HR 3.24, 95%CI 2.85–3.68. Treated subjects: vertebra vs. humerus/wrist: HR 1.80, 95%CI 0.83–3.91; hip vs. humerus/wrist HR 0.99, 95%CI 0.42–2.34

During the 24-month follow-up period, the mortality rate was 13.4% (95%CI 12.7–14.0), with older age, male gender, and IHCP implementation acting as independent predictors (Table 2). Compared to untreated participants, those receiving appropriate AFT reported a lower likelihood of dying (HR 0.55, 95%CI 0.37–0.82; *p* = 0.003), as well as those who appropriately initiate and persisted on treatment for 1 year (HR 0.18, 95%CI 0.10–0.34; *p* < 0.001), as did those receiving AFT treatment over 6 months from fracture (HR 0.05, 95%CI 0.02–0.14; *p* < 0.001). Interestingly, participants who were adherent to appropriate AFT had threefold lower mortality rates (2.1 deaths per 100 person-years, 95% CI 1.1–3.8) than those who initiate appropriate treatment but missed adherence (6.2 deaths per 100 person-years, 95%CI 4.2–9.2; HR 0.33, 95% CI 0.16–0.69, *p* = 0.003). Compared to participants with humerus or wrist fractures, those with hip (HR 1.98, 95%CI 1.74–2.25, *p* < 0.001) or vertebral (HR 1.40, 95%CI 1.19–1.66, *p* < 0.001) index fractures experienced a higher likelihood of dying. In a head-to-head comparison, subjects with hip confirmed higher mortality than those with vertebral fractures (*p* < 0.001). Treatment was recognized as an effect modifier in the association between the site of index FF and mortality (Fig. 2, *p* for interaction 0.003). The mortality rates per 100 person-years between treated and untreated participants were classified according to the type of index FF. Among untreated participants, mortality rates increased almost linearly from those with humerus or wrist, vertebra, and hip index FF (*p* < 0.001). In contrast, mortality rates by site of FF were dramatically lower in treated compared to untreated, and almost superimposed independent of the index FF (*p* = 0.190, Fig. 2). Building on a subgroup analysis by treatment, we showed a higher mortality likelihood among untreated compared to treated (Fig. 2).

## Discussion

This is the first study reporting on the effectiveness of an IHCP, based on the integrated FLS model and dedicated to the secondary prevention of FFs among high-risk subjects living in a large Italian healthcare district. We aimed to address the effect of IHCP on appropriate AFT initiation time and 1-year adherence, then exploring its impact on subsequent FFs and mortality up to 24 months from the index event.

Overall, the IHCP is an FLS-based integrated model tailored to the Italian NHS [12], integrating local authorities’ strategic plans, GPs valued in their role as clinical case managers, nurse case-managers, and BSs as focal points for second-third line treatments, and responsible for single or multiple FLS centers. The IHCP’s implementation showed a positive effect on AFT initiation rates, especially for the complete treatment among younger participants with humerus or wrist FFs, and improved 1-year adherence to complete treatment. Although initiation rates still remained low (13%), the majority (42%) of subjects initiated AFT within 6 months from the index event, with 54% showing 1-year adherence to AFT, and 40% to AFT plus vitamin D. Notably, the initiation rates were about 20% after vertebral fracture, remained about 10% after hip fracture, and registered a statistically significant increase of 20% among participants who underwent humerus or wrist FFs in the post-intervention phase. Secondary prevention pharmacological strategies upgraded according to patients’ risk profile, with prescription of fewer oral bisphosphonates and more subcutaneous or intravenous drugs, then suggesting effective patients’ co-managing through the hospital-community network. These findings mainly relied on GPs’ changed behaviors, which may reflect improved education and easier co-managing with BSs based on services integration. Indeed, subjects experiencing humerus or wrist fractures, which are frequently the first life-course fractures, are usually managed by GPs after an orthopedic evaluation. After IHCP implementation, more appropriate assessment and initiation rates were registered. Differently, individuals with vertebral and hip fractures still did not benefit from the IHCP. Many high-risk patients, especially those with hip fractures, received vitamin D and its metabolites as single treatment for secondary prevention. While the increased attention to first life-course FFs seems encouraging, there are not explanations for vitamin D as a secondary prevention strategy alone in high-risk patients.

The phenomenon highlights the need for more education about the efficacy and safety of AFT or the window of opportunity for tools helping to identify and monitor treatments’ renewal according to AIFA resolutions.

Despite improvement in appropriate treatment initiation, 1-year adherence remained substantially similar to that reported by the AIFA [16] and previously shown by FLS [9, 17, 18]. Surprisingly, 1-year adherence rates were superimposed among different types of index FFs, without a signal for better adherence among the younger group with humerus or wrist FFs. Despite existing recommendations and guidelines, the effects of the IHCP implementation remained limited with regard to the GPs’ role in the early identification of individual risk profiles and initiation of AFT tailored to disease severity and complexity. As a result, most high-risk patients, especially those with hip and vertebral fractures, remain unprotected and deprived of systematic assessment and follow-up for secondary falls and fracture prevention.

We acknowledge several factors contributing to these findings. First, we cannot exclude a negative influence of the COVID-19 pandemic, which occurred soon after the IHCP implementation, severely burdening the GPs’ activities, driving nurse case-managers’ resources to different settings, and impairing the expected adherence’s improvement, while the lockdown increased individuals’ disuse and fracture risk [19]. The overlap between the post-IHCP phase and the COVID-19 pandemic may have affected fracture risk assessment, treatment adherence, and access to healthcare services, especially among older adults and frail people. Then, the lockdown associated with it might have increased individuals’ fracture risk due to limited mobility and physical deconditioning, helping to disentangle the reasons for the increased re-fracture risk after the pathway implementation, as shown by other studies [20]. We sought that data still gathered since 2022 will definitely help to solve these hypotheses and demonstrate an effect on blunting the imminent risk of fracture [21].

As recommended by the FLS model [22], the nurse case manager has an essential role as a liaison professional between patients, specialists, and services, and as a focal point for delivering educational programs to patients and caregivers. This role promotes participants’ empowerment, optimizes the correct use of medications, and improves treatment adherence. Therefore, we argue that the study’s findings were limited by the lack of such resources in the post-intervention phase. In the absence of coordinated services for secondary prevention, GPs usually rely on the specialists’ judgment and recommendations, mainly those formulated by the orthopedic surgeons, physiatrists, and others primarily involved in the management of patients hospital-admitted due to major FFs. Third, we cannot ignore the role of AIFA boundaries, which regulates the balance between subjects’ risk profiles and drug reimbursement, in contributing to the low AFT initiation and adherence rates, especially among patients naive to treatments, both in the pre- and post-intervention phases. With these premises, the magnitude of the care gaps will further increase compared to that (71%) previously reported in the Italian sample participating in the SCOPE. Moreover, AIFA’s reimbursement constraints will impair the 20% gap reduction sustained by FLS implementation [9], as shown in other countries.

Concerning 2-year mortality, we showed a mortality rate of 13.4 per 100 person-years, which is consistent with previous findings [17, 23]. Compared to untreated subjects, those who initiated treatment within 6 months from the index event and persisted for 1 year on AFT experienced the lowest mortality likelihood. These findings confirm the beneficial effects of early treatments and adherence to AFT over the first year as previously shown [9, 23]. Participants adherent to appropriate AFT had threefold lower mortality rates than those who only initiated appropriate treatment; in both cases, death rates were dramatically lower than those found among untreated participants. Among untreated participants, those with hip fractures showed the highest mortality rates, double that observed among patients with vertebral FF, and almost fourfold higher than that of patients with humerus or wrist FF. Interestingly, AFT acts as an effect modifier of the association between hip FF and mortality, with a reduction in mortality likelihood almost superimposed to that associated with humerus, wrist, and vertebra FFs.

To our knowledge, this is the first study reporting such a significant impact of AFT on mortality after hip FF. A Portuguese FLS reported comparable results after 36 months of follow-up, although they did not reach statistical significance, possibly due to the relatively small sample [24]. The NoFRACT study did not show a protective effect of AFT on mortality after hip FF. However, although the wide cohort (*n* 16,326 subjects) included, hip fractures were the only inclusion criteria, limiting comparisons among different types of FFs [25]. A recent meta-analysis highlighted a reduction of mortality only within the first year after a hip FF, not extending over such a follow-up period [26]. Although initiation treatment rates in our study were almost superimposed to those previously reported by Silva [24] and Andreasen [25], the effect size of adherence against mortality was notably higher in our study than those previously reported. We hypothesize that a hospital-community integrated pathway of care, appropriately engaging services and professionals, may be a pivotal aspect of an FLS workflow, driving processes and leading outcomes. In our study, AFT adherence reflects GPs’ widespread involvement and engagement in monitoring patients’ treatment. Then, the adoption of the AIFA reimbursement algorithms for drug prescribing might also have contributed to improving therapy adherence after the IHCP model.

A bias effect due to indication should be acknowledged in this scenario, as physicians tend to treat patients who exhibit less frailty, less comorbidity, and less polypharmacy. Instead, patients with hip fracture are older and frailer than those affected by vertebra, humerus, or wrist FF, and these features can explain their higher likelihood to die, independent of the effect of AFT.

Indeed, older age and male gender confirmed their independent role as predictors of mortality, while a similar effect was shown by IHCP implementation. We hypothesize that the coincidence between the post-intervention phase and the COVID-19 pandemic may have influenced the findings.

Our study confirmed an overall re-fracture rate of 5.2 per 100 person-years as previously reported by FLS worldwide [9, 27]. Initiating AFT appropriately and remaining adherent to treatment over the first year tended to reduce participants’ re-fracture risk compared to untreated, and the protective effect was confirmed even when comparing those receiving AFT after 6 months from the index event with those untreated.

Appropriate initiation of AFT beneficially impacts adverse events [24, 28], including falls and fractures, even after hip FFs [23, 26], especially when secondary prevention starts in the early phase. Patients with hip fractures are those at the highest risk of re-fractures, accounting in our sample for 57.5% of all re-fractures, confirming previous evidence [29]. In addition, setting an IHCP at the district level was associated with a higher re-fracture likelihood in the post- compared to the pre-intervention phase. Finally, we acknowledge several limitations of the study. Retrieving data from an administrative database, as it is updated from data sent by minor and major hospitals, may result in stochastic reporting errors (e.g., missing ICD codes, under-reported traumas, duplicates), and lack of information regarding subject’s health, functional, socioeconomic conditions affecting individual well-being. Moreover, we do not know if participants enrolled in the study occurred as the first FF or more. The IHCP implementation in Italy is closely connected to AIFA reimbursement regulations; therefore, our findings might not apply to other healthcare systems (e.g., UK, Portugal, Scandinavia) [30], and differences in healthcare financing and drug accessibility could account for variations in the process and outcome metrics. Therefore, we cannot generalize the findings obtained from our study to other healthcare contexts, as they may offer different accessibility to AFT and reimbursement policies. Another limitation would be the study duration spanning across the Covid pandemic. However, in a sensitivity analysis excluding the year 2020, our conclusions on appropriateness and adherence did not vary.

The availability of administrative data is also the main strength of this study, as it gave us the most considerable possible number of subjects enrolled and their data, collected from all hospitals of Pavia province, both public and private, from physician prescriptions, pharmaceutical distribution of medication and drugs, re-fracture rates from Emergency Rooms and Orthopedic Units, and death reports. For these reasons, this database is the most comprehensive and authoritative source on the health situation in the Pavia province, thereby strengthening our results. The features and the outcomes associated with the setting of the community–hospital integrated model of care for secondary prevention for the inhabitants of the province of Pavia represent an added value for other areas and regions embedded within the Italian NHS. The findings emerging from the workflow implementation may provide valuable insights to inform future research in the field of secondary prevention of fragility fractures, even outside of the country boundaries.

## Conclusions

This study reports the effects of a structured model of IHCP from home to home broadening the FLS approach, involving GPs as clinical managers, nurses as case managers, and BSs as co-managers for high-risk patients. Despite the negative influence of the COVID-19 pandemic, we showed potential benefits of the IHCP on initiating appropriate complete AFT and fostering AFT adherence, then reducing mortality. However, patients’ outcomes demonstrated a poor momentum in closing the care gap of secondary prevention of FFs, bringing out educational needs and the opportunity for tools supporting the initiation and monitoring of appropriate AFT. More data are needed to confirm the effectiveness of the IHCP in Pavia’s district.

## Supplementary Information

Below is the link to the electronic supplementary material.

ESM 1 (PPTX 90.8 KB)

## Data Availability

The data that support the findings of this study are available from the corresponding author upon reasonable request.
