# Supplementary material for: Effectiveness of an integrated hospital-community care pathway for fragility fractures’ secondary prevention: the PROMOTER-II study
Source: Arch Osteoporos. 2025 Dec 16;21(1):13. doi: 10.1007/s11657-025-01642-0 (PMC12708766; doi:10.1007/s11657-025-01642-0)
Supplement: Supplementary file 1 — (PPTX 90.8 KB) [file 11657_2025_1642_MOESM1_ESM.pptx]

## Slide 1
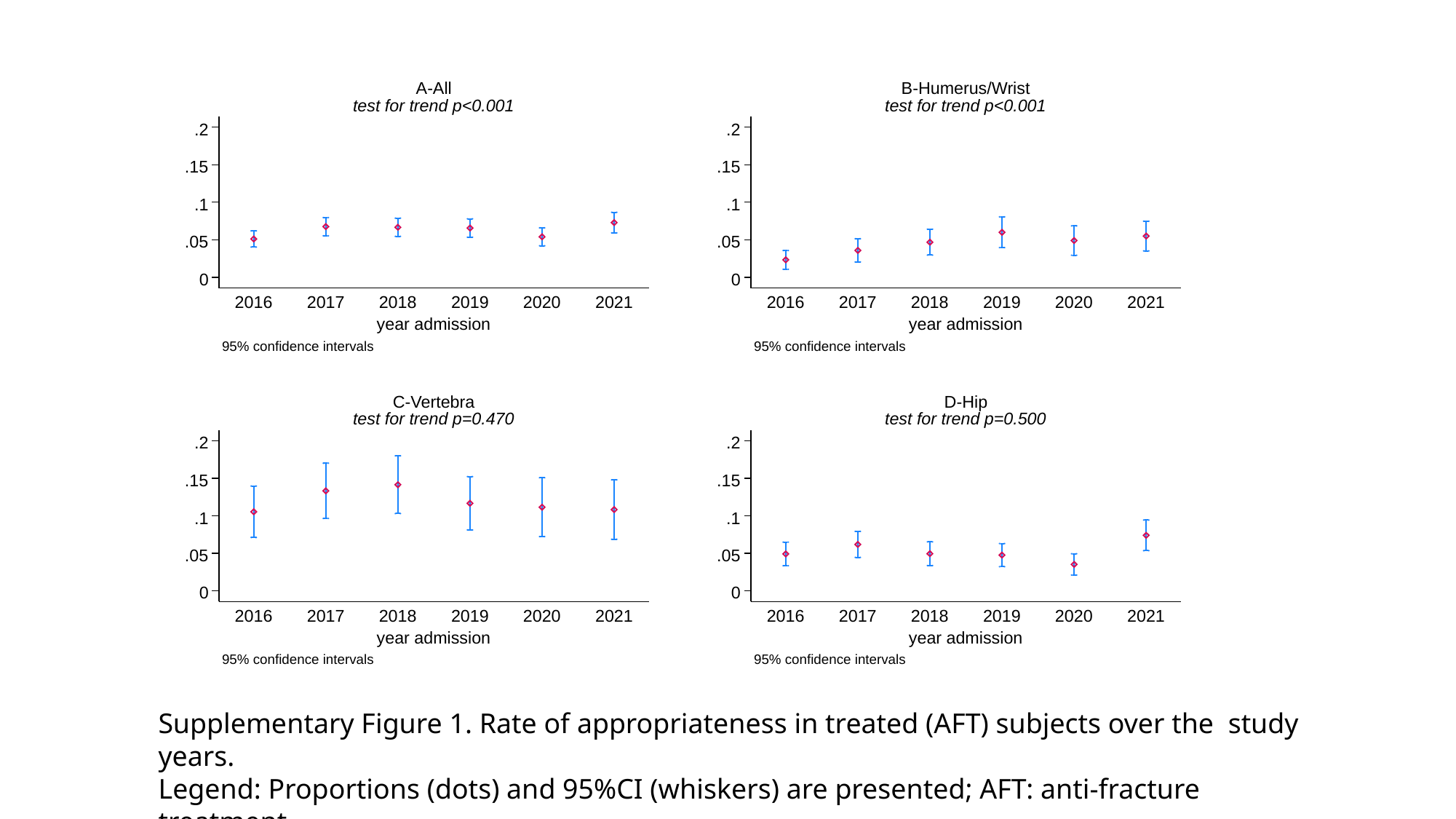

Supplementary Figure 1. Rate of appropriateness in treated (AFT) subjects over the study years.
Legend: Proportions (dots) and 95%CI (whiskers) are presented; AFT: anti-fracture treatment.

## Slide 2
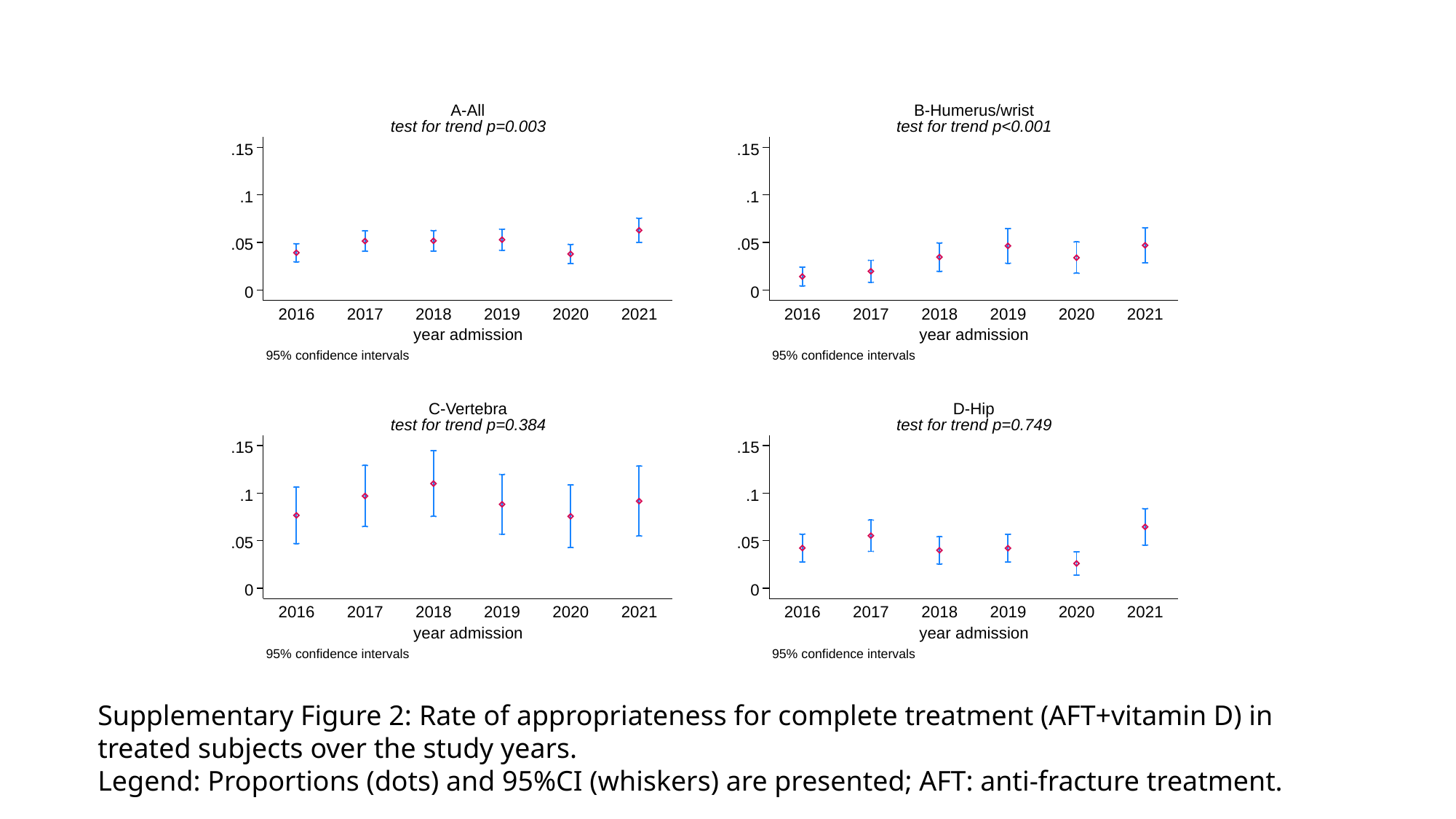

Supplementary Figure 2: Rate of appropriateness for complete treatment (AFT+vitamin D) in treated subjects over the study years.
Legend: Proportions (dots) and 95%CI (whiskers) are presented; AFT: anti-fracture treatment.

## Slide 3
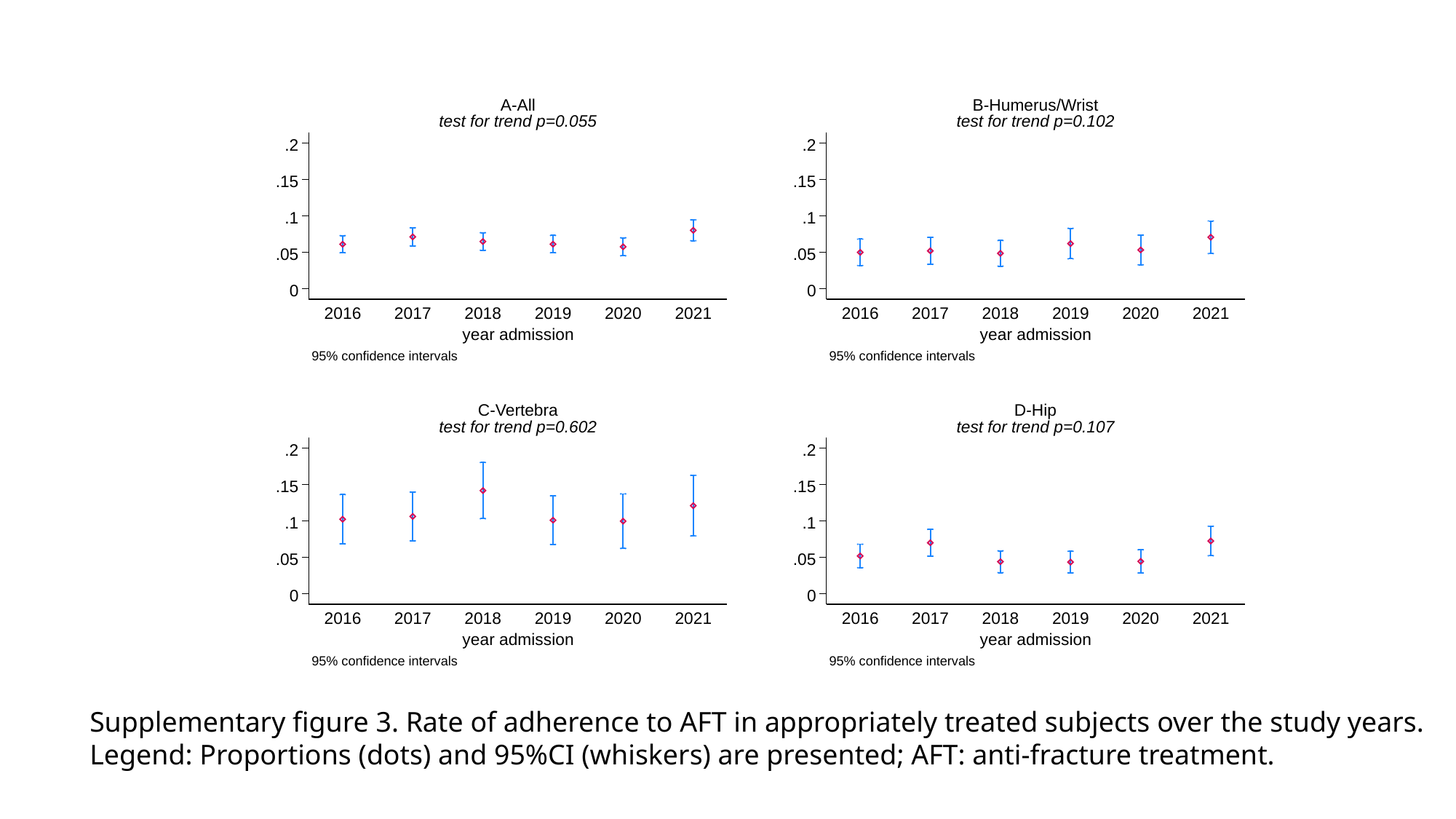

Supplementary figure 3. Rate of adherence to AFT in appropriately treated subjects over the study years.
Legend: Proportions (dots) and 95%CI (whiskers) are presented; AFT: anti-fracture treatment.

## Slide 4
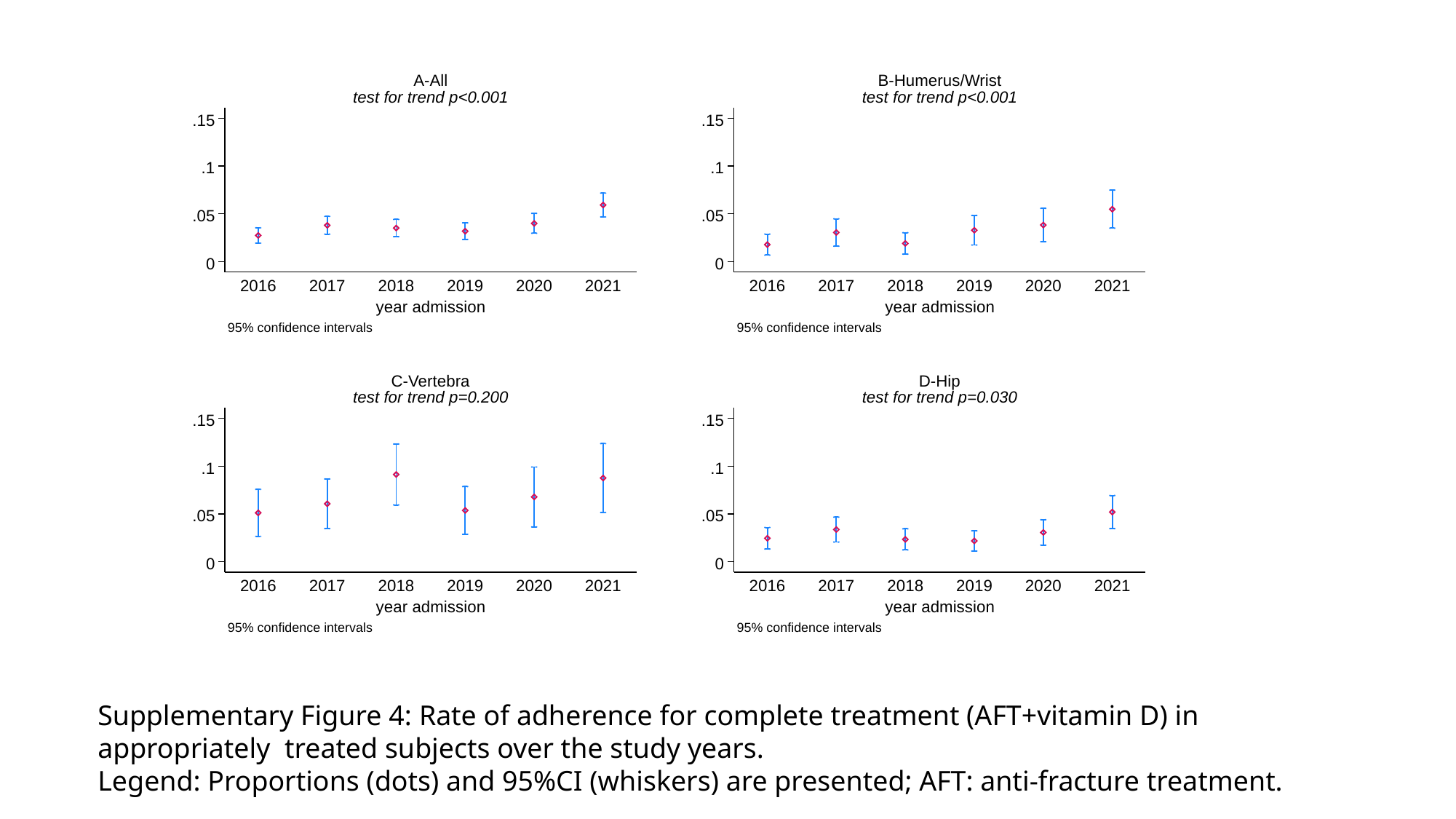

Supplementary Figure 4: Rate of adherence for complete treatment (AFT+vitamin D) in appropriately treated subjects over the study years.
Legend: Proportions (dots) and 95%CI (whiskers) are presented; AFT: anti-fracture treatment.
